# Effects of interval‐based inpatient treatment for anorexia nervosa: An observational study

**DOI:** 10.1002/brb3.2362

**Published:** 2021-09-20

**Authors:** Kathrin Peters, Adrian Meule, Ulrich Voderholzer, Elisabeth Rauh

**Affiliations:** ^1^ Schoen Clinic Bad Staffelstein Bad Staffelstein Germany; ^2^ Department of Pathopsychology University of Bamberg Bamberg Germany; ^3^ Department of Psychiatry and Psychotherapy University Hospital, LMU Munich Munich Germany; ^4^ Schoen Clinic Roseneck Prien am Chiemsee Germany; ^5^ Department of Psychiatry and Psychotherapy University Hospital of Freiburg Freiburg Germany

**Keywords:** anorexia nervosa, inpatient treatment, intermittent treatment, interval treatment, weight gain

## Abstract

**Objective:**

After inpatient treatment for anorexia nervosa (AN), many patients relapse and need to be readmitted. To obtain a sustained improvement, a pre‐planned multistep inpatient procedure might help to improve the patient's skills in dealing with symptoms and transdiagnostic problems, thus decreasing symptoms of AN. However, no data have been reported for such interval treatment yet. Therefore, this study examined effects of interval treatment in inpatients with AN.

**Method:**

Data of adult women with AN (*N* = 304) who received inpatient treatment and either received interval treatment (*n* = 179) or not (*n* = 125) were analyzed. Of these, 225 patients completed a follow up measurement after an average of 25 months. Treatment outcome variables were body mass index and subscales of the Eating Disorder Inventory‐2 at admission, discharge, and follow up.

**Results:**

Across measurements, the interval treatment group had larger increases in body mass index and larger decreases in drive for thinness and binge/purge symptoms than the no interval treatment group. These differences did not seem to be driven by longer treatment duration.

**Discussion:**

Our data suggest that interval treatment for AN is effective and may even be superior to conventional single inpatient treatment. Given the observational nature of this study, however, controlled studies are necessary to corroborate these findings.

## Graphical abstract

A multistep inpatient interval treatment might be helpful in the treatment of anorexia nervosa in order to improve the patient's skills in dealing with symptoms and transdiagnostic problems, thus reducing symptoms and relapses. This observational study is the first to examine an interval approach in the inpatient treatment of anorexia nervosa and indicates that patients treated with interval therapy showed a favorable outcome.

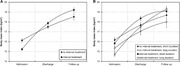

## 1 INTRODUCTION

With a point prevalence of 0.3% to 2.8% (Galmiche et al., 2019; Hoek, 2006; Hoek & van Hoeken, 2003), anorexia nervosa (AN) is a rare mental disorder, even within the at‐risk group of young women. At the same time, it is one of the most serious mental disorders with a standardized mortality rate of 5.5 to 5.9 (Arcelus et al., 2011; Fichter & Quadflieg, 2016). While it is often possible to increase body weight during inpatient treatment, many patients lose weight again in their home environment. Relapse rates—often associated with the need for inpatient (re)hospitalization—are in the range of 9% to 52% (in most studies above 25%; cf. Khalsa et al., 2017), with a meta‐analytically estimated mean rate of 31% after in‐ or outpatient treatment (Berends et al., 2018). The risk for relapse is particularly high within the first year after discharge from treatment, with remission rates only being between 13% and 50% (Berends et al., 2018; Brockmeyer et al., 2018; Fichter et al., 2017; Wonderlich et al., 2020). Furthermore, 20% to 30% of patients suffer from a persistent, often lifelong form of AN, which is often associated with multiple treatments (Eddy et al., 2017; Herpertz‐Dahlmann et al., 2018; Rydberg Dobrescu et al., 2020; Støving et al., 2011; Wonderlich et al., 2020).

Wonderlich et al. (2020) point out (a) that severe and enduring AN is often associated with short‐term weight gain (typically in higher levels of care), only to be followed by renewed weight loss and a “revolving door” pattern of admission and discharge, (b) that changes between health care providers can lead to contradictory treatments causing iatrogenic problems, and (c) that these processes might lead to demoralization. The risk of a renewed worsening may be partially due to the fact that treatments often achieve improvements in body weight (which reduces the somatic complexity and visibility of the disorder) whereas the psychopathological complexity persists (Fennig et al., 2017; Murray et al., 2018, 2019). Thus, patients would have to learn to decouple eating behavior from eating disorder‐related thoughts and fears outside of protected conditions during inpatient treatment. Specifically, patients should not adjust their eating behavior according to anorectic avoidance motives and weight desires. Instead, they should adjust their eating behavior according to a self‐care motive and accept the resulting weight trajectories.

This therapeutic rationale is conveyed to patients during inpatient treatment in our hospital (Schoen Clinic Bad Staffelstein, Germany). Yet, to further support the recovery process and anticipate activation of weight phobia in certain phases of recovery, we additionally offer interval treatment, which involves a planned alternation between inpatient therapy and transfer phases in the home environment (for further description see Method section). That is, rehospitalization does not take place only after aggravation but according to a pre‐defined schedule and in the same treatment facility, which might prevent the vicious circle pointed out by Wonderlich et al. (2020) and, therefore, chronification of AN. German treatment guidelines (Herpertz et al., 2018; Resmark et al., 2019) explicate that an interval approach for AN can be considered if achieving an adequate target weight (usually a body mass index [BMI] of 18.5 kg/m^2^) would require an exceedingly long inpatient treatment as is often the case with extremely underweight patients.

We conducted a detailed literature research in the databases Web of Science and PubMed as well as with Google Scholar, which did not yield any scientific articles that reported empirical data about the effectiveness of interval treatment for AN. The only report published in English that mentions interval therapy as part of a day‐patient treatment for eating disorders is a student paper from Sweden (Bonde & Härkönen, 2009). As interval treatment of AN has not been empirically examined yet, we analyzed data of female adults with AN with and without planned interval treatment to investigate the effectiveness of this approach.

We hypothesized that patients receiving interval‐based treatment would show better treatment outcomes (in terms of BMI and scores on the Eating Disorder Inventory‐2 [EDI‐2]) than patients receiving similar treatment—but without the in advance planned readmissions—in the same hospital. We further expected that this effect would not only be due to longer treatment duration but would also persist when analyses are controlled for total treatment duration.

Assumably, without interval treatment, risk of chronification and of exacerbation is higher because traditional inpatient treatments often end before significant aversive weight boundaries have been overcome and before relevant skills in dealing with symptoms and transdiagnostic problems have been acquired (e.g., regulation of aversive feelings and more or less intrusive thoughts; decoupling eating behavior from these feelings or thoughts; encouraging one's self‐esteem and assertiveness regardless of weight or performance). At unplanned readmissions due to acute exacerbation, patients usually experience further worsening because they need longer until they see the necessity for readmission. In combination with waiting time till admission, they have even greater weight loss. With new inpatient stays, often only the last weight at discharge is reached—resulting in the revolving door effect. This way, higher therapeutic goals (overcoming further aversive weight boundaries and, for example, self‐esteem and assertiveness difficulties) never get achieved.

In contrast, interval treatment limits home transfers to manageable periods of time during which regular mail reflection helps to stay focused on targets until treatment is continued at an agreed‐upon time to evaluate experiences and work specifically on identified difficulties. This way, it supports the patient especially during the vulnerable first year after discharge, with an intensity that cannot be performed by outpatient therapists alone. It also offers to accompany the patient during the anticipated trajectory of weight progress under regular and sufficient nutrition (which often does not end at BMI 18.5 kg/m^2^).

## 2 METHOD

### 2.1 Sample description and procedure

The study was conducted in accordance with the Declaration of Helsinki and approved by the Ethics Committee of the University of Bamberg, Germany (reference 2020‐1135). Written informed consent was obtained from all participants. Data of *N* = 304 women with AN who received inpatient treatment at the Schoen Clinic Bad Staffelstein (Germany) between 2016 and 2019 were analyzed. Specifically, all of these patients had one inpatient stay and, at discharge, were either assigned to receive further inpatient stays (interval treatment group) or not (no interval treatment group); a detailed description of the treatment and assignment follows below. Inclusion criteria were female sex, age between 18 and 55 years, and a length of stay of at least 21 days. Cases with shorter lengths of stay were excluded because these were premature discharges without sufficient treatment dose for evaluation and occurred before interval treatment could have been planned at all. These criteria were met by 304 patients, of which 166 (54.6%) patients were diagnosed with restrictive‐type AN (ICD‐10 code F50.00), 115 (37.8%) were diagnosed with binge/purge‐type AN (ICD‐10 code F50.01), and 23 (7.6%) were diagnosed with atypical AN (ICD‐10 code F50.1).

In mid‐2020, these patients were contacted for a follow up measurement that included both interview and questionnaire assessment. The interview was conducted by telephone using a specially developed structured guideline, after which patients received a link to the online questionnaires via e‐mail. Seventy‐nine patients (26.0%) could not be reached (one patient was deceased) or declined participation. Mean length of the follow up period (i.e., the time between discharge of the first inpatient treatment and follow up measurement) was *M* = 25.1 months (SD = 14.0, range 2–50). Thus, there were three measurements: admission and discharge of the first inpatient stay and the follow up measurement.

All patients received cognitive‐behavioral therapy in an inpatient treatment unit specialized on eating disorders. A weight gain of 500 to 1500 g per week was intended, in line with international treatment guidelines (Herpertz et al., 2018; Hilbert et al., 2017; Resmark et al., 2019). Average weight gain during the first stay of inpatient treatment was 0.59 kg/week (SD = 0.31). The therapeutic focus was on teaching the rationale of recovering (namely, eating regularly and sufficiently, that is, self‐caringly, and accepting the associated weight development) and on learning cognitive‐behavioral strategies (e.g., working with meal plans, weight mapping, exposure planning, ABC technique, defusion). In addition, therapy with interval treatments could be planned, which is a sequence of inpatient treatment phases and alternating home transfer phases. Interval treatment is indicated if (a) weight restoration within the anticipated range would require an exceedingly length of stay, (b) ensuring nutrition management in a self‐caring manner (and, thus, decoupling from eating disorder‐related thoughts and fears) requires training phases at home, and (c) the patient is motivated and committed to work on these goals. The agreement on an interval treatment is made jointly by the treatment team and the patient.

The tailored design of an interval plan is based on a standardized *weight mapping* (Peters & Rauh, 2021). This technique includes a retrospective, longitudinal evaluation of the nutrition and weight trajectory as well as an evaluation of the anticipated trajectory of weight progress under regular and sufficient nutrition. Considering the individual aversive weight boundaries, the scope of already acquired skills in dealing with symptoms and transdiagnostic problems, and contextual conditions (e.g., social or working conditions), future stays in the hospital are scheduled so that recovery is therapeutically accompanied in a demand‐actuated and forward‐looking manner. The aim of this planned intermittent treatment is to limit the extent of a possible weight loss between the stays and to evaluate the transfer in a timely manner. Learning objectives for the following interval stay should be derived from this to increase the patient's active skills in dealing with symptoms and transdiagnostic problems needed for recovering from AN. Interval treatment is, therefore, an individualized therapy planning with standardized methods. For this reason, duration and number of interval treatments and transfer phases differ between patients. Usually, the more severe a case of AN, the longer the inpatient stays and the shorter the stays at home environment. For a patient with a BMI of 14 kg/m^2^ at admission who anticipates weight regulation at BMI 20 kg/m^2^ as a result of self‐care eating behavior, traditional treatment would take a 36‐week stay for overcoming a difference of 6 kg/m^2^ (i.e., 18 kg) with 500 g/week. Following our rationale, an ideal course would be, for instance: First interval treatment for 18 weeks from 14 to 17 kg/m^2^, 8 weeks at home, second interval treatment for 12 weeks from 17 to 19 kg/m^2^, 16 weeks at home, third interval treatment for 8 weeks with non‐forced weight regulation from 19 to 20 kg/m^2^ and focusing on body acceptance, 8 months at home, fourth interval treatment for 2 weeks for assurance and final therapeutic concerns.

In our sample, interval group patients had, on average, three stays (intention‐to‐treat), and, on average, four stays in the subgroup that completed interval treatment regularly. At home, patients of both treatment groups are encouraged to practice acquired cognitive‐behavioral strategies (such as meal plans or others, as mentioned before), use outpatient care at place of residence (not performed by hospital as patients from all over Germany are treated at our unit) and—only as part of interval treatment—write reflection e‐mails regularly (which are answered only in a standardized manner, that is, with an acknowledgment of receipt and general encouragement, but without individualized therapeutic feedback).

Thus, in contrast to day‐patient treatment, interval therapy offers a more long‐term treatment with transfer phases that can last several weeks or months under everyday conditions (beyond short trials at evenings or weekends, as in the case of day‐patient treatment). Furthermore, due to the low prevalence of AN, specialized day clinic facilities are usually only feasible in larger cities for structural reasons. Consequently, for many patients, those facilities are not accessible in their region.

### 2.2 Measures

#### 2.2.1 Anthropometric and other information

Data about height and weight at admission and discharge were taken from the clinical records and used to calculate BMI. Other information such as age, comorbid mental disorders, previous inpatient treatments, and the number and duration of inpatient treatments during the study period were also taken from the clinical records. At follow up, body weight was assessed via interview and it was assessed whether and, if so, how long the patients had any other inpatient treatments at another hospital during the study period (i.e., between admission to the first inpatient stay and the follow up measurement). As this was a naturalistic observational study (and not a randomized controlled trial), however, the number and length of inpatient stays were heterogeneous. That is, some patients in the interval treatment group decided not to return for a second stay or did not complete all planned readmissions. In the no interval treatment group, some patients had unplanned readmissions (Table 1). Thus, to address the issue that the number and length of further inpatient stay between discharge from the first inpatient stay and the follow up measurement differed individually, we analyzed total treatment duration as the sum of treatment days of all inpatient stays (most of which were planned in the interval treatment group and all of which were unplanned in the no interval treatment group) during the study period.

**TABLE 1:** Descriptive statistics of study variables as a function of treatment groups

	*N*	No interval treatment group	Interval treatment group
Age (years)	304	*M* = 27.5 (SD = 9.60)	*M* = 25.6 (SD = 7.46)
Comorbid mental disorders^a^			
Total count	304	*M* = 0.98 (SD = 0.92)	*M* = 0.92 (SD = 0.95)
Any comorbidity	304	*n*/*N* = 83/125 (66.4%)	*n*/*N* = 121/179 (67.6%)
Previous inpatient treatments			
Total count	303	*M* = 1.81 (SD = 2.60)	*M* = 1.67 (SD = 2.91)
Any previous inpatient treatment	303	*n*/*N* = 71/125 (56.8%)	*n*/*N* = 108/178 (60.7%)
Inpatient treatments during study period			
Total count	225	*M* = 1.69 (SD = 1.30)	*M* = 2.98 (SD = 1.61)
Total treatment duration (days)	225	*M* = 97.0 (SD = 90.3)	*M* = 169 (SD = 87.0)
Length of follow up period (months)	225	*M* = 25.5 (SD = 14.7)	*M* = 24.9 (SD = 13.6)
Body mass index (kg/m^2^)			
Admission	304	*M* = 16.1 (SD = 2.36)	*M* = 15.2 (SD = 1.90)
Discharge	304	*M* = 17.5 (SD = 2.16)	*M* = 17.9 (SD = 1.56)
Follow up	222	*M* = 18.5 (SD = 2.96)	*M* = 19.2 (SD = 2.66)
Eating Disorder Inventory‐2 (mean scores)			
Drive for thinness			
Admission	223	*M* = 4.10 (SD = 1.50)	*M* = 4.35 (SD = 1.33)
Discharge	227	*M* = 3.52 (SD = 1.54)	*M* = 3.89 (SD = 1.33)
Follow up	190	*M* = 3.58 (SD = 1.50)	*M* = 3.30 (SD = 1.37)
Bulimia			
Admission	223	*M* = 2.31 (SD = 1.24)	*M* = 2.16 (SD = 1.26)
Discharge	227	*M* = 1.70 (SD = 0.89)	*M* = 1.49 (SD = 0.71)
Follow up	190	*M* = 2.12 (SD = 1.17)	*M* = 1.57 (SD = 0.74)
Body dissatisfaction			
Admission	223	*M* = 4.20 (SD = 1.24)	*M* = 4.29 (SD = 1.13)
Discharge	227	*M* = 4.02 (SD = 1.31)	*M* = 4.38 (SD = 1.23)
Follow up	190	*M* = 3.81 (SD = 1.26)	*M* = 3.81 (SD = 1.33)

^a^ The three most common comorbid mental disorders were depressive disorders (F32/F33; 61.5%), phobic anxiety disorders (F40; 6.3%), and post‐traumatic stress disorders (F43.1; 5.6%); all other comorbid mental disorders were less common (<5%).

#### 2.2.2 EDI‐2

Patients routinely completed the German version (Paul & Thiel, 2004) of the EDI‐2 (Garner, 1991) at admission and discharge and patients who participated at the follow up measurement completed the questionnaire again. The EDI‐2 has 91 items that are answered on a six‐point scale (1 = *never* to 6 = *always*). The questionnaire has 11 subscales. However, only three subscales assess eating disorder‐specific symptoms (drive for thinness, bulimia, body dissatisfaction) and, thus, only these subscales were used in the current analyses. Higher subscale scores indicate stronger drive for thinness, binge/purge symptoms, and body dissatisfaction, respectively. Internal reliability of the drive for thinness subscale ranged between *ω = .931 and .951*, internal reliability of the bulimia subscale ranged between *ω* = .883 and .918, and internal reliability of the body dissatisfaction subscale ranged between *ω* = .901 and .939 across the three measurements.

### 2.3 Data analyses

Treatment groups (no interval treatment vs. interval treatment) were compared regarding age, number of comorbid mental disorders, number of previous inpatient treatments, number of inpatient treatments during the study period, total treatment duration (i.e., the sum of treatment days of all inpatient treatments during the study period, see above), and length of the follow up period with independent samples *t*‐tests. We also tested with *χ* ^2^‐tests whether groups differed regarding any comorbid mental disorder and any previous inpatient treatment.

Changes in outcome variables across measurements as a function of groups were analyzed with growth curve analyses (Mirman, 2014) using the R‐package *lme4* (Bates et al., 2015). This analytic strategy has multiple advantages as compared to, for example, analysis of variance. For instance, it can handle missing data better (i.e., cases with missing data are not excluded but included in the maximum likelihood estimation), both categorical and continuous predictor variables can be analyzed, random effects can be specified, and both linear and nonlinear trajectories can be modeled. Specifically, nonlinear changes can be tested by adding a second‐order polynomial of the time term to the linear term. Here, we used orthogonal polynomials as has been recommended (Mirman, 2014). In addition, fixed effects of treatment groups on all time terms were added. Thus, to examine both linear and nonlinear changes across the three measurements as a function of treatment groups, we ran models that included the predictor variables time, time^2^, group, time × group, and time^2^ × group. The models also included random effects of patients on all time terms. Separate models were run for predicting BMI, drive for thinness, bulimia, and body dissatisfaction. To examine whether different time effects between treatment groups may simply be due to differences in total treatment duration, we also tested whether total treatment duration moderated any effects by testing the respective three‐way interactions. Parameter‐specific *p*‐values were calculated with the R‐package *lmerTest* (Kuznetsova et al., 2017). For all analyses, *p*‐values < .05 were considered as indicating a significant effect.

## 3 RESULTS

### 3.1 Group differences

Groups did not differ in age, number of comorbid mental disorders, number of previous inpatient treatments, and length of the follow up period (all *p*s > .05, *d*s < .23, Table 1). They also did not differ in having any comorbid mental disorder (*χ* ^2^_(1)_ = 0.05, *p = .827*, *φ* = 0.01) and any previous inpatient treatment (*χ* ^2^_(1)_ = 0.46, *p* = .500, *φ* = 0.04, Table 1). The interval treatment group had a higher number of inpatient treatments during the study period (*t*_(223)_ = 6.23, *p* < .001, *d* = 0.86) and, hence, a longer total treatment duration (*t*_(223)_ = 5.87, *p* < .001, *d* = 0.81) than the no interval treatment group (Table 1). Of note, however, is that 16.9% (*n*/*N* = 24/142) of patients in the interval treatment group did not return for a second stay and thus, only had one inpatient treatment. In turn, 33.7% (*n*/*N* = 28/83) of patients in the no interval treatment group had more than one inpatient treatment.

### 3.2 Changes in BMI as a function of groups

**FIGURE 1:**
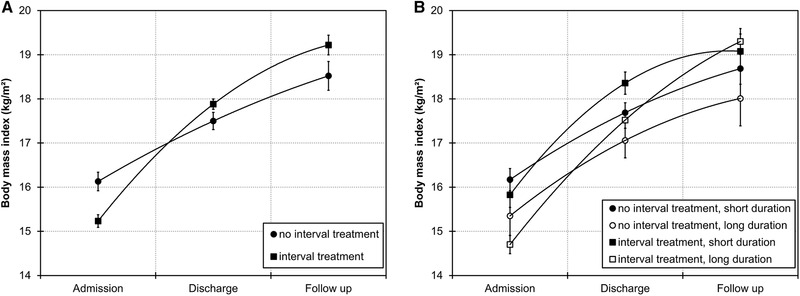
Mean body mass index (BMI) with second‐order polynomial fit lines. Error bars represent the standard error of the mean. Panel (a) displays BMI as a function of treatment groups. Panel (b) displays BMI as a function of treatment groups and total treatment duration. Short and long total treatment durations are based on a median split for which mean total treatment duration was 57 and 224 days in the no interval treatment group and 85 and 214 days in the interval treatment group. Note, however, that analyses are based on the continuous variable of total treatment duration and not based on group categorization, which only serves the purpose of visualizing the interaction effect

When predicting BMI, the time^2^ × group interaction was significant (estimate = −0.36, SE = 0.15, *p* = .020), indicating that slopes differed in shape between groups. As can be seen in Figure 1a, the no interval treatment group had a nearly linear increase in BMI whereas the interval treatment group had a nonlinear and steeper increase in BMI across measurements. When testing the moderating role of total treatment duration, however, the time^2^ × group × total treatment duration interaction was also significant (estimate = 0.004, SE = 0.002, *p* = .020), indicating that slopes differed in shape as a function of both groups and total treatment duration. As can be seen in Figure 1b, the two slopes in the no interval treatment group are nearly parallel, indicating that BMI increased equally across measurements regardless of treatment duration. In the interval treatment group, however, the slope for patients with a short treatment duration is nonlinear, indicating an attenuated BMI increase from discharge to follow up while the slope for patients with a long treatment duration is nearly linear, indicating larger BMI increases from discharge to follow up. In other words, patients in the no interval treatment group who had more than one inpatient treatment did not profit from these subsequent, unplanned treatments as much as patients in the interval treatment group did profit from subsequent, planned treatments.

### 3.3 Changes in EDI‐2 scores as a function of groups

**FIGURE 2:**
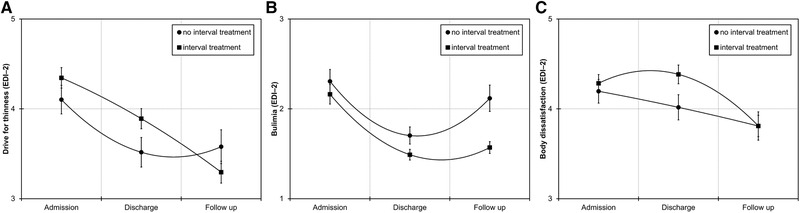
Mean subscale scores of the Eating Disorder Inventory‐2 (EDI‐2) with second‐order polynomial fit lines. Error bars represent the standard error of the mean. Panel (a) displays mean scores of the drive for thinness subscale, panel (b) displays mean scores of the bulimia subscale, and panel (c) displays mean scores of the body dissatisfaction subscale as a function of treatment groups

When predicting drive for thinness scores, the time^2^ × group interaction was significant (estimate = −0.26, SE = 0.10, *p* = .013), indicating that slopes differed in shape between groups. As can be seen in Figure 2a, the no interval treatment group had a nonlinear decrease in drive for thinness with no change between discharge and follow up while the interval treatment group had a linear decrease in drive for thinness with continued decreases in scores between discharge and follow up. When testing the moderating role of total treatment duration, the time^2^ × group × total treatment duration interaction was not significant (estimate = 0.001, SE = 0.002, *p* = .472). When including total treatment duration as covariate only (i.e., without including interaction effects with this variable), the time^2^ × group interaction was still significant (estimate = −0.22, SE = 0.11, *p* = .044).

When predicting bulimia scores, the time^2^ × group interaction was not significant (estimate = −0.08, SE = 0.08, *p* = .347), indicating that slopes did not differ in shape between groups. However, when removing the nonlinear time term from the model, the time × group interaction was significant (estimate = −0.25, SE = 0.11, *p* = .025), indicating that slopes differed in steepness. As can be seen in Figure 2b, both groups had nonlinear changes in bulimia but scores increased from discharge to follow up in the no interval treatment group and remained stable in the interval treatment group. When testing the moderating role of total treatment duration, the time × group × total treatment duration interaction was not significant (estimate = −0.001, SE = 0.001, *p* = .401). When including total treatment duration as covariate only (i.e., without including interaction effects with this variable), the time × group interaction was still significant (estimate = −0.43, SE = 0.12, *p* < .001).

When predicting body dissatisfaction scores, the time^2^ × group interaction was significant (estimate = −0.28, SE = 0.10, *p* = .007), indicating that slopes differed in shape between groups. As can be seen in Figure 2c, the no interval treatment group had a nearly linear decrease in body dissatisfaction whereas the interval treatment group had a nonlinear decrease with no change from admission to discharge. When testing the moderating role of total treatment duration, the time^2^ × group × total treatment duration interaction was not significant (estimate = 0.0004, SE = 0.002, *p* = .769). When including total treatment duration as covariate only (i.e., without including interaction effects with this variable), the time^2^ × group interaction was still significant (estimate = −0.29, SE = 0.11, *p* = .009).

## 4 DISCUSSION

The current study examined treatment effects in adult inpatients with AN in a naturalistic longitudinal study. We hypothesized that patients receiving interval treatment would show a better outcome after discharge than patients without planned interval stays, although patients in the latter group also had readmissions (all of which, however, were unplanned). This hypothesis was supported when examining changes in BMI and was partially confirmed when examining changes in EDI‐2 scores.

### 4.1 Changes in body weight

Both groups showed significant increases in BMI from admission to follow up, which indicates that patients profited from the taught therapeutic rationale and acquired cognitive‐behavioral strategies after discharge. However, groups differed in magnitude of weight changes with the interval treatment group showing larger weight gain than the no interval treatment group. This effect emerged although the interval treatment group started at a lower BMI at admission, as shown in Figure 1a. Lower BMI at admission is commonly associated with poorer outcome (e.g., Fichter et al., 2017; Vall & Wade, 2015). Thus, the better outcome of the interval treatment group cannot be attributed to these group differences at admission.

On average, patients in the interval treatment group increased their weight by 4.0 kg/m^2^ (compared to 2.4 kg/m^2^ in the no interval treatment group, Table 1) from admission to follow up. Since groups differed in total treatment duration, larger weight gain in the interval treatment group may potentially be due to this longer and more intensive treatment. However, our analyses suggested that this does not seem to be the case. Patients in the interval treatment group with a longer total treatment duration did indeed have a steeper BMI increase from discharge to follow up than those with a shorter total treatment duration. In the no interval treatment group, however, total treatment duration did not affect weight changes (as indicated by the parallel lines in Figure 1b). Thus, it appears that patients in the no interval treatment group did not profit from subsequent, unplanned readmissions as much as patients in the interval treatment group did profit from additional, planned readmissions.

### 4.2 Changes in cognitive‐behavioral eating disorder symptoms

Drive for thinness decreased similarly in both groups from admission to discharge but only patients with interval treatment further improved from discharge to follow up. Controlling for total treatment duration did not influence this effect. Similarly, binge/purge symptoms decreased from admission to discharge in both groups from admission to discharge but only interval treatment patients’ scores remained stable whereas no interval treatment patients showed an increase in binge/purge symptoms between discharge and follow up. Again, this effect was not influenced when controlling for total treatment duration. Thus, interval treatment might help to acquire better and more lasting transfer skills whereas the temporary decrease in the no interval group at discharge may be an effect of the protecting conditions within the hospital. The fact that no further decline in binge/purge symptoms was found in the interval treatment group may be due to floor effects as symptom severity was already non‐pathological at discharge. Changes in body dissatisfaction also differed between groups such that groups had similar levels of body dissatisfaction at admission and follow up but the interval treatment group had higher scores than the no interval treatment group at discharge. While higher body dissatisfaction at discharge in the interval treatment group might be a result from the larger weight gain in that group, it might also be that higher levels of body‐related concerns at discharge have increased motivation to agree to interval treatment.

Overall, findings based on the EDI‐2 subscales are in accordance with other studies (Fennig et al., 2017; Murray et al., 2018, 2019), which conclude that traditional AN treatments have relatively strong immediate effects on behavior and, consequently, the somatic state of patients (e.g., BMI), but only weak effects on psychopathological variables. This may be one reason why severe underweight can—on average—be overcome but body weight usually remains in the upper underweight or lower normal‐weight range after treatment. Instead, offering alternating intervals of treatment and transfer phases at home seems to lead to larger and long‐lasting effects not only in body weight but also in cognitive‐behavioral eating disorder symptoms.

### 4.3 Strengths and limitations

The current study has several strengths. We investigated the course of AN after inpatient treatment in a realistic clinical setting with a relatively large sample size. To our knowledge, this is the first study that reports empirical data about the effectiveness of interval treatment in AN. As we report intention‐to‐treat analyses, effects of interval treatment are rather underestimated because effects include data of patients who did not finish their interval treatment or even had no interval therapy at all (17%). However, this also highlights that further research on how to improve adherence to interval treatment is necessary.

Limitations of this study include that interpretation of results is limited to female adults with AN and, thus, may not apply to adolescent or male patients. Although length of the follow up interval did not differ between groups (see Table 1) and, thus, is unlikely to have affected results in the current study, the range of the follow up interval is quite broad and a more homogeneous time interval for follow up in future studies is desirable.

A general methodological limitation of observational studies is that patient behavior between inpatient stays is not controlled. Therefore, future studies should control for outpatient aftercare. In the future, it should also be systematically recorded how many patients disregarded therapy requests, especially with regard to interval treatment.

A further limitation of the current study is that body weight at follow up was based on self‐reports, which may be biased. Yet, women with AN are extremely accurate when self‐reporting their weight. For example, self‐reported weight has been found to be more accurate in women with AN than in normal‐weight and overweight women (Engstrom et al., 2003). Although it has been found that they slightly overestimate their weight, this overestimation is on average less than 1 kg (Ciarapica et al., 2010; McCabe et al., 2001; Meyer et al., 2009). According to another study, there is relatively accurate self‐report in individuals with AN and slight underestimation in remitted AN (Wolfe et al., 2013). Thus, it is unlikely that using self‐report of current weight at follow up substantially affected results of the current study.

Crucially, this was not a randomized controlled trial, that is, assignment to interval versus no interval treatment was based on clinical decision and request by patients, which might be influenced by factors such as motivation or compliance. Thus, it cannot be determined which effects this non‐randomized selection had on treatment adherence and outcome. Therefore, randomized controlled trials are urgently needed to corroborate results from this naturalistic observational study.

### 4.4 Clinical implications

A crucial question about interval treatment is whether it is feasible and can also be implemented in countries other than Germany. One might think that, due to the German healthcare system, transferability to other countries in which inpatient treatment might be less supported may be restricted. For example, it has been noted previously that inpatient treatment for AN in Germany is longer and more intensive than most of the structured treatment available in many parts of the world (Attia, 2014). In interval treatment group, however, the total duration of treatment (days in our hospital or other hospitals since first admission at our unit) was 169 days on average. Other studies in adult samples described mean length of stays with around 90 days (Fichter et al., 2017; Schlegl et al., 2014), 90 inpatient days, and 50 days of day treatment (Dalle Grave et al., 2020), 123 days (Roux et al., 2016), 156 days (Danielsen et al., 2020) and 184 days (Goddard et al., 2013). However, none of these studies included additional treatment duration in later stays because of readmissions as we did in our study. Considering this, interval treatment seems to be within the range of the usual lengths of treatments (at least in some parts of the world). Thus, we would argue that interval treatment may seem like a time‐consuming and costly approach but when considering the many unplanned readmissions that are often required in the treatment of AN, it may turn out as a time‐ and cost‐efficient alternative to traditional treatment approaches.

## 5 CONCLUSION

This study indicates that interval treatment could be an effective strategy for treating AN in adults and might even be a superior alternative to conventional procedures with only one hospital stay. Yet, controlled studies are necessary to confirm superiority and to examine factors such as treatment adherence, feasibility issues, and comparisons to other treatment approaches.

### PEER REVIEW

The peer review history for this article is available at https://publons.com/publon/10.1002/brb3.2362

## Data Availability

The data that support the findings of this study are available from the corresponding author upon reasonable request.
